# Hyperthyroidism Secondary to Disseminated Mucormycosis in a Child with Acute Lymphoblastic Leukemia: Case Report and a Review of Published Reports

**DOI:** 10.1007/s11046-012-9584-1

**Published:** 2012-09-25

**Authors:** Ninela Irga, Wojciech Kosiak, Radoslaw Jaworski, Jolanta Komarnicka, Dorota Birkholz

**Affiliations:** 1 Department of Pediatrics, Hematology, Oncology and Endocrinology, Medical University of Gdansk, Debinki 7, 80-211 Gdansk, Poland; 2 Department of Pediatric Cardiac Surgery, Pomeranian Centre of Traumatology, 1-6 Nowe Ogrody, 80-803 Gdansk, Poland; 3 Department of Clinical Microbiology, Clinical Center of Medical University of Gdansk, Debinki 7, 80-211 Gdansk, Poland

**Keywords:** Mucormycosis, Leukemia, Thyroiditis, Hyperthyroidism

## Abstract

Thyroiditis due to fungal infection is an extremely rare cause of hyperthyroidism. The most common etiological factor of thyroiditis is *Aspergillus*. Infections due to members of the *Mucorales* have been an increasing clinical problem in recent years, and the prognosis in generalized infections due to those fungi is usually very poor. No hyperthyroidism in a child with thyroiditis due to mucormycosis has been reported in the literature so far. We describe a clinical course of generalized mucormycosis with thyroid involvement in a 12-year-old girl treated for acute lymphoblastic leukemia. The child underwent a hyperthyroidism connected with thyroid involvement due to a fungal process. The diagnosis was based on the clinical signs, laboratory findings and typical ultrasound scan; however, later attempt to amplify the fungi DNA from the tissue block has failed. The child died because of multiorgan failure due to general fungal infection 49 days after the invasive fungal infection was diagnosed. The generalized mucormycosis is always connected with poor prognosis and the mortality is high.

## Introduction

Hyperthyroidism (HT) due to thyroiditis is rarely observed in children treated for neoplastic diseases. One of the causes of HT may be an infectious process in the thyroid gland. Fungi, most commonly *Candida* and *Aspergillus* species, belong to infectious factors of HT [1–3]. Literature gives only few reports on thyroid involvement in the history of mucormycosis, even though a constant increase in invasive fungal infections (IFIs) due to members of Mucorales has been observed in recent years, especially in children treated because of proliferative diseases of the hematopoietic system [4, 5]. Mucormycosis may be a reason of hyperthyroidism in the history of thyroiditis. No hyperthyroidism in a child with thyroiditis due to mucormycosis has been so far reported in the literature.

## Case Report

**Fig. 1:**
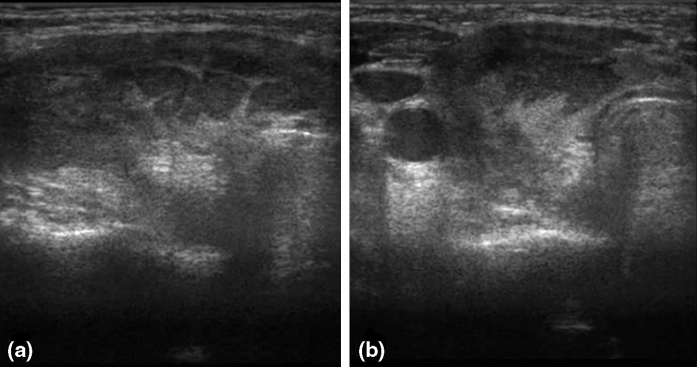
Right thyroid lobe: **a** longitudinal scan—diffused hypoechoic area with hyperechoic linear fibrous septa and focal isoechoic areas; **b** transverse scan—decreased echo of thyroid gland with isoechoic areas in the central part of right lobe and isthmus

**Fig. 2:**
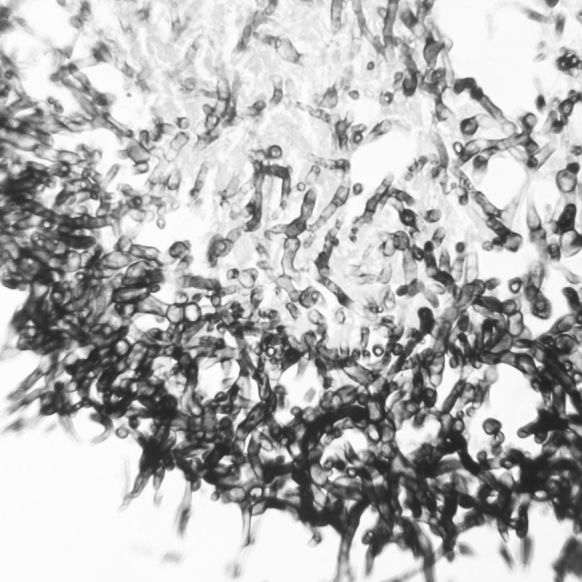
Fungal hyphae with wide angle branching in thyroid tissue. Gomori methenamine silver stain

A 12-year-old girl was hospitalized due to acute lymphoblastic leukemia (ALLIC 2002 protocol, intermediate risk group). Routine imaging examinations before treatment introduction (brain magnetic resonance imaging, abdominal ultrasound scan as well as neck and thyroid ultrasound scan) showed no abnormalities. Agranulocytosis with neutrophils count below 200 per μL was observed since the introduction of antineoplastic therapy (prednisone 60 mg/m^2^, vincristine, daunorubicin, L-asparaginase). On the 21st day of therapy, the general condition of the patient deteriorated and inspiratory–expiratory dyspnoea was observed. Computed tomography of the chest showed diffuse inflammatory infiltration in the majority of the right lung and free fluid in the right pleural cavity. The child required mechanical ventilation for 7 days. Since the etiological factor of the pulmonary infiltration remained unknown and because there was no clinical improvement in spite of the introduced antibacterial (cefepime 100 mg/kg daily replaced with meropenem 60 mg/kg daily and vancomycin 40 mg/kg daily) and antifungal treatment (voriconazole 12 mg/kg daily), we decided to perform an exploratory pleural puncture. Direct microscopy of the pleural fluid was negative. Pleural fluid was incubated at 37 °C the first 24 h and then at room temperature. The outgrowing colonies on Sabouraud agar and Columbia agar with 5 % blood were fast growing, floccose and white in color (becoming darker after several days). Microscopic examination in lactophenol blue stain revealed wide non-septate hyphae and sporangiophores with short branches bearing spherical sporangia with columellae. Collars were evident after rupture of the sporangia and apophysis was absent. The strain was mesophilic. The described characters identified the strain as *Mucor* sp. The strain was susceptible to amphotericin B (MIC 0.5) and resistance to caspofungin (MIC 32) and voriconazole (MIC 32). Posaconazole tests were not available in that period in our laboratory. Combined antifungal treatment was introduced—amphotericin B lipid complex (10 mg/kg daily) and posaconazole (600 mg daily). After extubation attempt, we observed a change in the voice timbre, which was related to intratracheal intubation and mechanical ventilation. Three weeks after the combined antifungal treatment, the patient demonstrated alarming clinical symptoms such as increasing anxiety, psychomotor agitation, psychotic symptoms and sleeplessness. The psychiatrist who consulted the child diagnosed psychotic syndrome and prescribed a phenothiazine antipsychotic. Four days later, we observed septic fever which did not respond to antipyretics. There were numerous loose stools and the girl reported spasmodic abdominal pains. All microbiologic examinations of the blood, urine and stool as well as viral examination of the stool turned out to be negative. On examination, we observed tachycardia (heart rate >180 per min) and arterial hypertension with a high systolic and diastolic amplitude. The thyroid gland was enlarged and tender on examination, with no flare or increased temperature of the skin over the gland. Hormonal evaluation confirmed the tentative diagnosis of hyperthyroidism (TSH—0.088 μU/mL, fT4—26.56 pmol/L, fT3—3.95 pmol/L). The level of antithyroid antibodies was unremarkable, including the level of TSH receptor antibodies. Hyperthyroidism was diagnosed. An ultrasound scan of the thyroid gland was also performed, and it revealed signs of an intense inflammatory process with an increased flow (Fig. 1). An uneventful ultrasound-guided fine-needle aspiration biopsy of the thyroid gland was carried out under general anesthesia. The histopathological examination showed signs of an intense inflammatory process. The direct Gomori methenamine silver-stained preparation revealed broad irregular, rarely septated fungal hyphae typical for *Mucorales*; the hyphae were rarely branching with wide angle and bulbous dilatations (Fig. 2). Intravenous infusions with thiamazole derivative and propranolol were introduced in the treatment and gave a rapid regression of hyperthyroidism, including the psychotic symptoms. Control hormonal evaluation proved to be normal 18 days after the introduction of antithyroid treatment (TSH—4.59 μU/mL, fT4—12.96 pmol/L, fT3—2.28 pmol/L). Despite the complex antifungal therapy, further progression of mucormycosis was observed. Even though a pulmonectomy was carried out, further spread of the infection to the right kidney and the contralateral lung was seen. The child died because of multiorgan failure due to general fungal infection 49 days after the invasive fungal infection was diagnosed. No autopsy examination was performed.

After 2 years, we tried to amplify the fungi DNA from tissue in reference mycology center. Formalin-fixed, paraffin-embedded (FFPE) tissue was sent to the Mycology Laboratory at the New York state Department of Health, Albany, New York, for molecular identification of etiologic agent identified by histopathology. The tissue was processed for DNA extraction using QIAamp DNA FFPE tissue (Qiagen, Valencia, CA) kit as per the manufacturers’ instructions, with minor modifications. After tissue lysis step, glass beads were added; and the mixture was disrupted in a cell disrupter for 30 min, followed by DNA extraction using reagents provided in the kit. The conventional PCR was carried out to amplify internal transcribed spacer 1 (ITS1) and ITS2 regions and the 5.8S ribosomal DNA (rDNA) region by using universal primers ITS1 and ITS4. The ITS2 region was simultaneously amplified by using universal primers ITS3 and ITS4. No PCR amplicon was detected with either set of primers. These results indicated that either there was complete lack of fungal DNA in the extracted tissue DNA or the amount of fungal DNA was not sufficient to be amplified by conventional ITS-PCR. It is also important to note that paraffin-embedded tissue has historically been viewed as an insensitive source for PCR assay [6].

## Discussion

In the available literature, there is no case report of a child with HT due to mucormycosis. The diagnosis of HT is based on the presence of clinical signs such as fever, tachycardia and heart palpitations, nausea, diarrhea, muscle weakness, fatigue and insomnia. However, in children who undergo antineoplastic therapy, such symptoms most commonly suggest the onset of general bacterial or fungal infection. Therefore, the diagnosis of HT in the described patient was not easy for oncohematologists. It is also important that the child presented no previous signs of hyperthyroidism, which would make the diagnosis easier. The ultrasound scan of the thyroid gland performed during the initial diagnosis of leukemia showed no abnormalities. It should also be stressed that psychotic symptoms came long before the other symptoms of HT. That is why it is extremely important for pediatric hematologists to include HT in the differential diagnosis of those symptoms.

Involvement of the thyroid gland in fungal infections is rarely diagnosed; such diagnosis is often reached *postmortem* [7, 8]. The most common etiological factor of thyroiditis is *Aspergillus.* Infections due to members of the *Mucorales* have been an increasing clinical problem in recent years, and the prognosis in generalized infections due to those fungi is usually very poor [9–11]. The most common manifestations of mucormycosis in Europe were pulmonary (30 %), rhinocerebral (27 %), soft tissue (26 %) and disseminated disease (15 %) [9]. The involvement of the thyroid gland in the history of mucormycosis is described in the literature extremely seldom (Table 1). The *intra vitam* diagnosis of thyroiditis due to a member of the *Mucorales* in our patient was based on the clinical signs, hormonal evaluation, ultrasound scan and histopathological examination. We supposed thyroiditis due to *Mucor* sp. in the result of generalized fungal infection with the beginning in the right lung (positive culture from the pleural fluid). The introduction of the appropriate therapeutic procedure in our patient led to regression of HT symptoms. Unfortunately, despite the complex antifungal therapy and pulmonectomy, further spread of the infection to other organs was observed. Such an unfavorable course of generalized mucormycosis is reported by numerous authors [5, 12]. We also considered thyroidectomy concomitant with pulmonectomy. However, due to the serious general condition of the patient and the severity of the elective pulmonectomy—after consulting anesthesiologists—the patient was disqualified from thyroidectomy.

**Table 1:** Thyroid gland involvement in mucormycosis [3, 7, 13–18]

Author	Publication year	Patient’s age (years)	Underlying condition	Pathogen	Diagnosis	Outcome
Chiba et al. [13]	1990	–	Acute myelogenous leukemia (AML)	*Cunninghamella bertholletiae*	Autopsy	Died
Vessely et al. [14]	1996	42	Rapidly progressive glomerulonephritis and pulmonary failure	Mucormycosis	Biopsy	Alive
Solano et al. [15]	2000	59	Immunocompetent	*Saksenaea vasiformis*	Autopsy	Died
Fujii et al. [7]	2003	48	Acquired immunodeficiency syndrome (AIDS)	Mucormycosis	Autopsy	Died
Kubota et al. [16]	2003	70	Myelodysplastic syndrome (MDS)	Mucormycosis	Autopsy	Died
Saikia et al. [3]	2007	53	2 months after a renal transplantation	Mucormycosis	Autopsy	Died
Minet et al. [17]	2009	25	9 months after a pulmonary transplantation	*Absidia corymbifera* ^a^	Biopsy	Alive
Mayayo et al. [18]	2011	50	Acute lymphoblastic leukemia (ALL)	*Cunninghamella bertholletiae*	Autopsy	Died
Mayayo et al. [18]	2011	42	Acute myelogenous leukemia (AML)	*Cunninghamella bertholletiae*	Autopsy	Died
Present case	2011	12	Acute lymphoblastic leukemia (ALL)	Mucormycosis	Biopsy	Died

^a^Pathogen original description, the currently accepted name of *Absidia corymbifera* is *Lichtheimia corymbifera*

The child died due to general fungal infection because of multiorgan failure. The generalized mucormycosis is still connected with poor prognosis and the mortality remains high.
